# Clinical application of a novel endoscopic mask: a randomized controlled, multi-center trial in patients undergoing awake fiberoptic bronchoscopic intubation

**DOI:** 10.1186/s12871-017-0370-y

**Published:** 2017-06-15

**Authors:** Tianxiao Zou, Zhenling Huang, Xiaoxue Hu, Guangyu Cai, Miao He, Shanjuan Wang, Ping Huang, Bin Yu

**Affiliations:** 1 Department of Anesthesiology, Tongji Hospital affiliated to Tongji University, Shanghai, China. NO.389, Xincun Road, Putuo District, Shanghai, China; 2 Department of Anesthesiology, Renji Hospital affiliated to School of Medicine, Shanghai Jiao Tong University, Shanghai, China; 3 Department of Anesthesiology, Shanghai Guanghua Hospital, Shanghai, China

**Keywords:** Awake fiberoptic bronchoscopic tracheal intubation, Endoscopic mask, Randomized controlled

## Abstract

**Background:**

Awake fiberoptic bronchoscopic tracheal intubation is usually regarded as an effective method in the management of predicted difficult airway. Hypoxia during awake nasal fiberoptic bronchoscopic intubation leads to discontinuation of the procedure, prolonged manipulation time and increased risk of severe complications. The main aim of the study was to test whether the novel endoscopic mask is helpful for hypoxia during the intubation.

**Methods:**

This was a randomized, controlled, multi-center study. 55 patients were recruited, but one patient was lost to follow-up. Finally, 54 patients (19 man and 35 women) were analyzed. After entering the operating room, nasal catheter oxygen-providing was given in the control group, and the treatment group received endoscopic mask oxygen-providing, with a flow rate of 3 L/min, lasting into the end of the intubation. Primary outcomes included mean arterial pressure, heart rate, minimum pulse oxygen saturation and incidence of pulse oxygen saturation ≤ 90%. Secondary outcomes included number of intubation attempts and time to intubation. All outcomes were finally measured.

**Results:**

Minimum pulse oxygen saturation during awake nasal fiberoptic bronchoscopic tracheal intubation was significantly higher in the endoscopic mask intubation group (91.7% ± 4.7%) than that the nasal catheter intubation group (87.6% ± 8.2%, *P* = 0.031. Furthermore, the incidence of pulse oxygen saturation ≤ 90% was significantly lower in the endoscopic mask intubation group (20.0%, 5/25) than that in the nasal catheter intubation group (51.7%, 15/29, *P* = 0.037). But mean arterial pressure of during intubation was significantly higher in the endoscopic mask group (100.0 ± 13.3 vs 90.3 ± 21.8, *P* = 0.049). In addition, there were no differences in the number of intubation attempts (*P* = 0.45) or time to intubation between the two groups (*P* = 0.38).

**Conclusions:**

The endoscopic mask was safely used in awake fiberoptic bronchoscopic tracheal intubation, with advantages of stable blood pressure and potential prevention of desaturation. Beginners for the intubation procedure and patients at high risk of hypoxia could benefit from the use of the endoscopic mask.

**Trial registration:**

Trial registration: www.chictr.org.cn. Registration No.: ChiCTR-TRC-13004086. Date of Registration: 8th, Sep, 2013.

## Background

Awake fiberoptic bronchoscopic tracheal intubation is usually regarded as an effective method in the management of predicted difficult airway. However, primary complications during fiberoptic bronchoscopic tracheal intubation, such as epistaxis, laryngeal trauma, laryngospasm, aspiration, respiratory depression, etc., still trouble doctors and patients [1, 2]. Many kinds of agents are used for decreasing discomfort of patients during awake fiberoptic intubation, including propofol, dexmedetomidine, midazolam, diazepam, meperidine, remifentanil, fentanyl [3–5]. But fentanyl and propofol can cause respiratory depression [2, 6], which may lead to failure of intubation and additional intubation. Midazolam combined with dexmedetomidine or remifentanil can also result in hypoxemia during fiberoptic intubation [3]. Additional intubation could increase the probability of epistaxis, laryngeal trauma, laryngospasm and aspiration, which may lead to high failure rate and mortality of intubation.

**Fig. 1:**
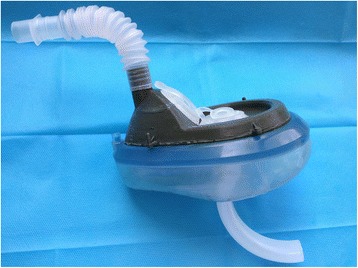
The endoscopic nasal mask used in this study

Nasal catheter is the traditional method of oxygen-providing during fiberoptic intubation. We want to develop other methods for sufficient oxygen supply, thus reducing the number of intubation attempts and time-consuming of intubation, and finally improving the success rate and safety during fiberoptic intubation. The purpose of this study was to compare the effectiveness of nasal catheter and novel endoscopic nasal mask (Fig. 1; PRC patent, ZL2012 1 0286504.5) in oxygen-providing during awake nasal fiberoptic bronchoscopic tracheal intubation in patients with difficult airway undergoing surgery.

## Methods

This randomized, controlled, multi-center trial was approved by the ethics committee of Shanghai Tongji Hospital, Shanghai Guanghua Hospital and Shanghai Renji Hospital. The trial was registered in the Chinese Clinical Trial Registry (http://www.chictr.org.cn; registration no.: ChiCTR-TRC-13004086; principal investigator: Bin Yu; date of registration: 8th, Sep, 2013). The subjects were recruited from patients who underwent surgery with predicted difficult airway. Inclusion criteria included patients with mouth disorder, respiratory obstruction, stiff neck, cervical spine fracture dislocation, anterior cervical scar contracture, high laryngeal prominence, short neck and micrognathia. Patients with airway tumor, airway anatomic abnormalities, airway injuries, coagulation abnormalities, taking anticoagulant drugs, skull fracture, giant aneurysm, acute laryngitis, pregnancy and lactation women were excluded.

Before participating in the study, subjects signed written informed consent. Eligible subjects were adult patients with predicted difficult airway. All patients were fasted for 12 h before undergoing awake nasal fiberoptic bronchoscopic tracheal intubation. 0.1 g Phenobarbital and 0.5 mg atropine was given by intramuscular injection before intubation. Electrocardiographic data, heart rate, pulse oxygen saturation, respiration and blood pressure were monitored during the procedures.

**Fig. 2:**
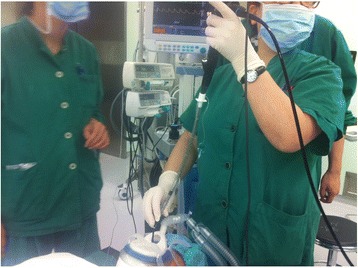
The endoscopic nasal mask being used during awake nasal fiberoptic bronchoscopic tracheal intubation

Fiberoptic bronchoscope was passed into nose via the nasal opening of the endoscopic nasal mask (Fig. 2). During the procedure, the oxygen-flow rate was set at 3 L/min. The respiratory interface, connected to the oxygen supply equipment, was to be used for first-aid measures if hypoxia occurred.

The study was a completely-randomized-design study, and subjects were randomly divided into nasal catheter group and endoscopic mask group. The essential information of the subjects who underwent awake nasal intubation was recorded, including sex, age, weight, height, important history and basal pulse oxygen saturation before intubation. Unilateral intravenous access was established via a peripheral vein in the upper limb. Electrocardiographic data, pulse oxygen saturation, respiration, heart rate and blood pressure were monitored. Subjects were randomized to a nasal catheter intubation group and an endoscopic mask intubation group, with both groups receiving oxygen at a flow rate of 3 L/min. In all subjects, intubation was performed after the injection of 2 ml of 2% lidocaine via a thyrocricoid puncture, the bilateral application of ephedrine and furacilin nasal drops, the administration of topical anesthesia in the mouth and throat, and the intravenous injection of 0.05 mg fentanyl and 1.5 mg/kg propofol. Transnasal tracheal intubation was guided by fiberoptic bronchoscope under mild sedation with spontaneous breathing. Anesthesia was induced with propofol and maintained with sevoflurane and propofol. Subjects were sent to the PACU for observation for 1 h after the surgery or to the intensive care unit, and were followed up 24 h later.

Primary outcomes were mean arterial pressure, heart rate, minimum pulse oxygen saturation and incidence of pulse oxygen saturation ≤ 90%. Secondary outcomes included number of intubation attempts and time to intubation.

The incidence rate of respiratory inhibition in patients who underwent awake nasal intubation with endoscopic mask was 0% in the pilot study [7, 8], while the same rate with the use of nasal catheters was 12.5% in a previous study [8]. A minimum of 24 subjects needed to be enrolled in the awake nasal intubation group, with an estimated subject attrition rate of 15%, and so the final requirement was for 28 patients. Continuous variables were compared using the t-test or Mann–Whitney U test, depending on the distribution of the data. Categorical variables were compared using the Pearson chi-square test or Fisher exact test. Statistical significance was assumed at a two-sided *P* value of <0.05. The results were expressed as mean with standard deviation(SD). Statistical analyses were performed using SPSS version 20.0 (SPSS Inc., Chicago, IL).

## Results

**Fig. 3:**
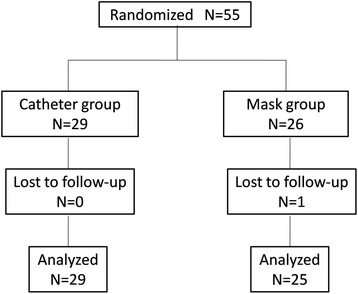
Flow chart of participant selection for awake nasal fiberoptic bronchoscopic tracheal intubation

A total of 55 patients met the inclusion criteria for awake nasal fiberoptic bronchoscopic tracheal intubation and were randomized (Fig. 3). One patient was lost to follow-up. Thus, finally, the data of 54 patients were analyzed (29 in the nasal catheter intubation group and 25 in the endoscopic mask intubation group), conforming to the minimum sample size required. The baseline characteristics of the patients have been reported in Table 1.

**Table 1:** Demographic and clinical characteristics of patients who underwent awake nasal fiberoptic bronchoscopic tracheal intubation group

	Endoscopic mask group (*n* = 25)	Nasal catheter group (*n* = 29)
Age,yr. (SD)	59.2 (13.5)	59.3(11.5)
Male (%)	8 (32)	11 (37.9)
Weight, kg (SD)	67.6(11.9)	64.6(12.6)
ASA risk score (%)		
I	6 (24)	10 (34.5)
II	19 (76)	19 (65.5)
Basal SPO2, % (SD)	97.4 (1.7)	97.4 (1.8)

Notes: *SPO2* pulse oxygen saturation, *ASA* American Society of Anesthesiologists

**Fig. 4:**
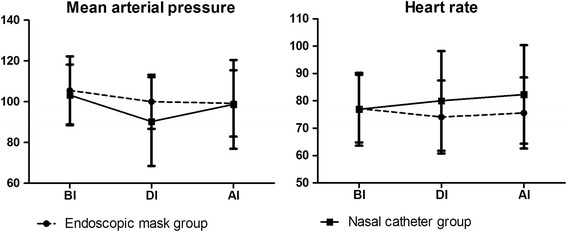
Mean arterial pressure and heart rate for nasal catheter and endoscopic mask intubation groups. BI = Before intubation; DI = During intubation; AI = After intubation

The trajectories of mean arterial pressure and heart rate in both groups are shown in Fig. 4. The heart rate showed no differences during the entire procedure of intubation, but the mean arterial pressure of during intubation was significantly higher in endoscopic mask group (100.0 ± 13.3 vs 90.3 ± 21.8, *P* = 0.049). The minimum pulse oxygen saturation during awake nasal fiberoptic bronchoscopic tracheal intubation was significantly higher in the endoscopic mask intubation group (91.7% ± 4.7%) than that in the nasal catheter intubation group (87.6% ± 8.2%, *P* = 0.031; Table 2). Furthermore, the incidence of pulse oxygen saturation ≤ 90% was significantly lower in the endoscopic mask intubation group (20.0%, 5/25) than that in the nasal catheter intubation group 51.7%, 15/29, *P* = 0.037). In addition, there were no differences in the number of intubation attempts (*P* = 0.45) and time to intubation (*P* = 0.38) between the two groups.

**Table 2:** Outcomes of awake nasal fiberoptic bronchoscopic tracheal intubation

	Endoscopic mask group (*n* = 25)	Nasal catheter group (*n* = 29)	*P* Value
Minimum SPO2, % (SD)	91.7 (4.7)	87.6 (8.2)	0.031
Incidence of SPO2 ≤ 90%, NO. (%)	5 (20.0)	15 (51.7)	0.037
Number of intubation attempts, NO (SD)	1.4 (0.7)	1.2 (0.5)	0.45
Time to intubation, s (SD)	179.6 (207.9)	136.1 (144.4)	0.38

## Discussion

We found the mean arterial pressure in the endoscopic mask group was more stable than in the nasal catheter group during intubation.The endoscopic mask sealed the face and respiratory interface connected to the ventilator allowing oxygen entering the respiratory tract through mouth and nose, thus providing continuous oxygen. The nasal opening allowed simultaneous nasal passage of the bronchofibroscope and endotracheal tube, as well as the oral opening. Once desaturation occurs during the intubation procedure, immediately high oxygen flow or sac pressure oxygen inhalation through the respiratory interface could be used as first-aid measures sequentially without removing the bronchofibroscope or the endotracheal tube. Compared to the common mask, the endoscopic mask was more convenient for tracheal intubation thanks to additional nasal opening and oral opening with continuous oxygen supplement. Moreover, the novel endoscopic mask could be used in other medical procedures, such as gastroscopy, tracheoscopy, choledochoscopy and enteroscopy.

The incidence of desaturations rates <90% in published awake fiberoptic bronchoscopic tracheal intubation studies is between 8 and 14.7% [3, 8–10], when oxygen is supplied by standard nasal cannula. Woodall et al. investigated impact of hypoxia on healthy unsedated volunteers undergoing awake fiberoptic bronchoscopic tracheal intubation, and found the incidence of desaturation below 80% was 1.5% when receiving oxygen via standard low-flow nasal cannula [11]. The use of sedatives could increase the incidence of respiratory depression, and the application of lidocaine to the upper airway has been found to reduce dynamic inspiratory airflow during the intubation procedure [12]. The elderly who suffered from cardiorespiratory diseases were more sensitive to hypoxidosis and had a higher risk of rapid oxygen desaturation.

A high-flow humidified transnasal oxygen-delivery system was developed to manage predicted difficult airway during awake fiberoptic bronchoscopic tracheal intubation [13]. The system was observed well tolerated in patients retaining spontaneously breathing undergoing awake fiberoptic bronchoscopic tracheal intubation and augmented oxygenation, thus potentially preventing desaturation arising. Standard nasal cannula can only supply up to 36% fractional inspired oxygen, but the system could provide the patient continuously with 100% fractional inspired oxygen during awake fiberoptic bronchoscopic tracheal intubation.

The Patil mask was designed to overcome the problem of ventilation during fiberoptic intubation [14, 15]. There was only one case of using the Patil mask during gastrointestinal endoscopy. Sundar et al. reported the case of a 36-year-old man with dysphagia in whom upper gastrointestinal endoscopy was performed with the aid of a Patil mask [16]. Unfortunately, this patient suffered from Duchenne muscular dystrophy and could not move any muscle in his body, except to speak. Furthermore, he required continuous positive airway pressure on account of inadequate respiratory power, which could lead to severe hypoxia during upper gastrointestinal endoscopy. Considering the risks of using muscle relaxants in patients with Duchenne muscular dystrophy and the damage that can be caused by the direct insertion of an endotracheal tube through the vocal cords without muscle relaxants, a non-invasive ventilation method was used to facilitate the gastroscopy procedure. With the Patil mask used as an aid for the procedure, the patient’s vital signs remained stable, and he recovered from the anesthesia and left the PACU uneventfully. Actually, our novel endoscopic nasal mask may be another good choice for this special patient and one of objectives of the endoscopic mask was designed to solve the complicated situation like this case. However, we did not design different sizes of mask based on our novel mask fitting for demand of endoscopy in infants and young children, and we will try to improve in this area.

## Conclusions

In conclusion, we recommend the endoscopic mask as an oxygen-providing device for patients with predicted difficult airway during awake fiberoptic bronchoscopic tracheal intubation. Stable blood pressure and potential prevention of desaturation could be the advantage of the endoscopic mask, which could provide better teaching and training opportunities for anesthetists especially for beginners of the intubation procedure. We also advocated its use in other medical procedures, such as gastroscopy, tracheoscopy, choledochoscopy and enteroscopy.
